# Extracytoplasmic Function σ Factors as Tools for Coordinating Stress Responses

**DOI:** 10.3390/ijms22083900

**Published:** 2021-04-09

**Authors:** Rubén de Dios, Eduardo Santero, Francisca Reyes-Ramírez

**Affiliations:** Departamento de Biología Molecular e Ingeniería Bioquímica, Centro Andaluz de Biología del Desarrollo, Universidad Pablo de Olavide/Consejo Superior de Investigaciones Científicas/Junta de Andalucía, 41013 Sevilla, Spain; rdebar@upo.es (R.d.D.); esansan@upo.es (E.S.)

**Keywords:** extracytoplasmic function σ factors, stress response, signal transduction, anti-σ, transcription

## Abstract

The ability of bacterial core RNA polymerase (RNAP) to interact with different σ factors, thereby forming a variety of holoenzymes with different specificities, represents a powerful tool to coordinately reprogram gene expression. Extracytoplasmic function σ factors (ECFs), which are the largest and most diverse family of alternative σ factors, frequently participate in stress responses. The classification of ECFs in 157 different groups according to their phylogenetic relationships and genomic context has revealed their diversity. Here, we have clustered 55 ECF groups with experimentally studied representatives into two broad classes of stress responses. The remaining 102 groups still lack any mechanistic or functional insight, representing a myriad of systems yet to explore. In this work, we review the main features of ECFs and discuss the different mechanisms controlling their production and activity, and how they lead to a functional stress response. Finally, we focus in more detail on two well-characterized ECFs, for which the mechanisms to detect and respond to stress are complex and completely different: *Escherichia coli* RpoE, which is the best characterized ECF and whose structural and functional studies have provided key insights into the transcription initiation by ECF-RNAP holoenzymes, and the ECF15-type EcfG, the master regulator of the general stress response in Alphaproteobacteria.

## 1. Introduction and Aim of this Review

Bacteria, like all other living organisms, have adapted and evolved to inhabit a specific environment, tuning their physiology and metabolism to the pervading conditions provided by their habitat. However, bacteria also need to be prepared to face constant changes in their environment. These fluctuations may lead to important deviations from the optimal growth conditions for a species, threatening bacterial survival, and are known as stress conditions. Stress may be generated by environmental changes and by the biochemical processes performed in the cell, although it may also appear as nutrient starvation or interactions with a host. Furthermore, a stress-inducing condition is subjective and specific for each species, so that different bacterial representatives may differ in their response to the same stimulus.

When the conditions shift to a less favorable situation, bacteria fight for survival by activating a number of mechanisms and regulatory circuits whose main effect is the mitigation, or removal, of the stress. The gene products synthesized to overcome stress conditions are variable depending on the stress. For example, temperature shifts may lead to the production of chaperones that aid in protein folding and proteases to remove misfolded proteins [1]. These shifts can also result in the production of proteins involved in maintaining the fluidity and functionality of the cell membrane [2]. Oxidative stress can be overcome by expressing catalases or peroxiredoxins [3,4], which catalyze the conversion of ROS (reactive oxygen species) into harmless compounds before they can damage cell structures. Alternatively, the response can result in the production of components that repair already damaged proteins, such as thioredoxins and glutaredoxins [5,6]. The host immune system, which is activated upon bacterial infection, can also be considered a stress for a pathogen and can result in activation of mechanisms to evade the host defenses [7]. These are the so-called bacterial stress responses, which comprise sensing of the stress and the ultimate activation of the resistance system through a signal transduction process. Actually, any type of bacterial signal transduction mechanism (reviewed in [8]) may be used to exert these responses, as long as its goal is to prepare the cell to face a threatening situation. However, from a classical point of view, bacterial signaling is performed by one- and two-component systems (OCSs and TCSs, respectively), which may introduce the added complexity of a phosphorelay when more than one phosphotransfer step occurs. Recently, Ser/Thr kinase systems started to gain relevance in the bacterial world, since they were originally thought to have a minor role compared to their functions in *Eukarya* [9]. This occurs despite sharing a common evolutionary origin [10] and contrasting with the His/Asp kinase/phosphotransfer mechanisms on which most TCSs rely.

Besides these mechanisms to regulate the activation of stress responses, alternative σ factors can also be used by bacteria. σ factors are subunits of the RNA polymerase (RNAP) holoenzyme essential for transcription initiation, due to their role in guiding the catalytic core of the RNAP in promoter recognition. Moreover, their specific helicase activity on the -10 box facilitates the formation of the transcription bubble through formation of the open complex. Apart from the vegetative σ^70^ factor, which ensure the bulk of the transcriptional control of house-keeping genes, alternative σ factors control the expression of a number of specific genes (regulons) with a defined collective function, which may go beyond the stress response. In the early 1990s, certain representatives of this group caught the attention of molecular microbiologists (reviewed in [11]) because of their small size. They comprise the essential domains allowing RNAP interaction, promoter-binding and initiation activity. They were named extracytoplasmic function σ factors (ECFs), since at that time they were thought to respond exclusively to environmental cues. Indeed, most of them are activated by external stimuli, although they can also respond to cytoplasmic signals. Due to their diversity and the relative simplicity of their mechanism of action, they have stood out as a versatile and powerful bacterial tool to efficiently activate stress responses [12].

Since their discovery, much effort has been focused on unraveling all biological aspects of ECFs. These include their role in bacterial physiology, their evolution and classification, the regulation of their production and activity, the stimuli that activate them and their target genes, their structural features and their transcription initiation mechanisms. Our knowledge on ECFs is continually growing, and excellent recent reviews have covered the findings of this broad ranging research field. Nevertheless, it is of interest for the scientific community to place our knowledge on ECFs into a broader context by discussing the various aspects of their biology, thus regarding their overall role in cellular physiology from a holistic viewpoint. For this reason, in this review, we comprehensively cover the most prominent aspects of the biology of ECFs, such as their role in the response to stress, how they are regulated, their classification, their mechanism of action and the future perspectives we consider to be important in future research on these regulators.

## 2. Bacterial σ Factor Families

**Figure 1:**
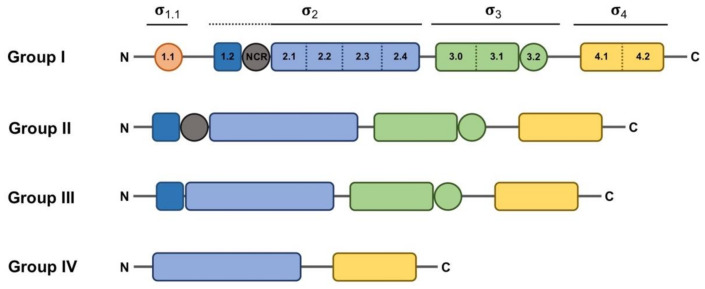
Schematic representation of the domain and subdomain architecture present in the different groups of the σ^70^ family. Regions that do not have a direct interaction with DNA appear as circles. Domains σ_1.1_, core σ_2_, σ_3_ and σ_4_ appear in orange, blue, green and yellow, respectively. Subdomains σ_1.2_ and σ_NCR_ (non-conserved region), which are considered part of the σ_2_ domain but are not conserved in all σ^70^ family groups, are depicted in dark blue and gray, respectively, and indicated with a dotted line. The extent of the rest of the σ domains is indicated with solid lines.

When considered from a structural point of view, σ factors can be divided in two main, structurally unrelated families: one large group includes the primary σ^70^ family, and the other much smaller group includes the RpoN family (classically known as σ^54^ or σ^N^), [13,14]. The RpoN family is generally represented by one member per bacterial genome. Although the difference between these two families is not the main focus of this review, it is nevertheless important to stress that RpoN acts in concert with ATP-dependent activators to help unwind the double-stranded DNA (dsDNA) near the transcription start site (TSS) to initiate transcription. In contrast, RNAP holoenzymes formed with σ^70^ family members melt the DNA near the TSS in an ATP-independent manner and without the aid of additional regulatory proteins [15]. The σ_2_ domain is able to melt the DNA at the -10 box once the RNAP holoenzyme is correctly positioned thanks to the DNA-binding activity of σ_4_ and σ_3_ domains to other promoter regions, such as the -35 box and the extended -10 region, respectively [16]. These structural domains, plus another additional domain with non-essential activity for promoter binding (σ_1.1_), form the basis of the typical structure feature of the primary σ^70^ factor, ascribed to Group I (Figure 1). σ factors belonging to Groups II–IV are considered alternative, because they are usually not essential for cell viability and may diverge from Group I in the presence of certain domains. Group II σ factors, with one well-characterized example in *Escherichia coli* RpoS, conserve the same domain architecture as the primary σ^70^ factor (RpoD in *E. coli*) except for the absence of the region σ_1.1_ [17], and their absence does not result in lethality. Thus, they are generally defined as non-essential σ^70^ homologs. Members of Group III, such as *E. coli* RpoH and FliA, essentially conserve domains σ_2_, σ_3_ and σ_4_ [17]. Although σ_3_ and σ_4_ bind different promoter regions, they are considered redundant, since the RNAP holoenzyme can bind and orientate efficiently in the absence of one of these domains or one of their DNA-binding determinants [16]. ECFs comprise Group IV with their structure being determined by the presence of only domains σ_2_ and σ_4_ separated by a linker shorter than 50 residues. Actually, “Group IV” would be a more appropriate term to describe these σ factors, since it is meanwhile clear that some ECFs respond to intracellular stimuli [13].

Classification of σ^70^ family members has relied on the presence of various well-conserved protein domains and subdomains (Figure 1). However, in some cases, the boundaries of this classification are diffuse, as some proteins may act as σ factors despite showing low similarity or a different organization, or vice versa, some proteins may have a structure resembling a σ factor whilst not functioning as such. For instance, a group of proteins encoded by *Clostridium* species that control the production of extracellular toxins, such as TcdR or BotR, function as σ factors at the biochemical level, and even show somewhat recognizable σ_2_ and σ_4_ domains [18,19]. However, the marked sequence divergence from Group IV representatives prevents a clear-cut classification into that group. This led to the proposition of these proteins as members of a novel category (Group V), branching from Group IV. Another example of these hard-to-define σ elements is the SigO-RsoA system, formerly YvrI-YvrHa [20,21]. In this pair, the σ_4_ and σ_2_ domains are split into separate SigO and RsoA proteins, respectively, and together are able to initiate the transcription of the *oxdC-yvrL*, *yvrJ* and *yvrI-yvrHa* operons. Finally, the response regulator PhyR, whose function will be explained below in more detail, has a N-terminal σ-like domain [22], and although this domain reveals a correct ECF structure, the regions responsible for the interaction with the promoter sequence and the core RNAP appear degenerated, preventing it acting in transcription initiation. Instead, upon activation of the PhyR protein, this domain serves to titrate the anti-σ factor NepR from its cognate ECF EcfG, thus triggering the alphaproteobacterial General Stress Response (GSR).

## 3. Transcription Initiation: σ^70^ Factor vs. ECFs

Clearly, the regulation of σ factor availability and/or activity is a powerful means by which gene expression can be regulated, particularly because different RNAP holoenzymes can initiate the transcription of diverse gene clusters at the same time by swapping only one subunit. Thanks to many structural and functional studies deciphering the catalytic mechanism of RNA polymerase, many aspects related to both promoter recognition and dsDNA strand separation are known for the primary σ^70^ factor [23,24,25,26,27,28,29,30,31,32,33,34]. More recently, a number of studies have shed light on the structures and mechanistic differences of transcriptional initiation driven by ECFs in comparison with the σ^70^ factor [35,36,37].

**Figure 2:**
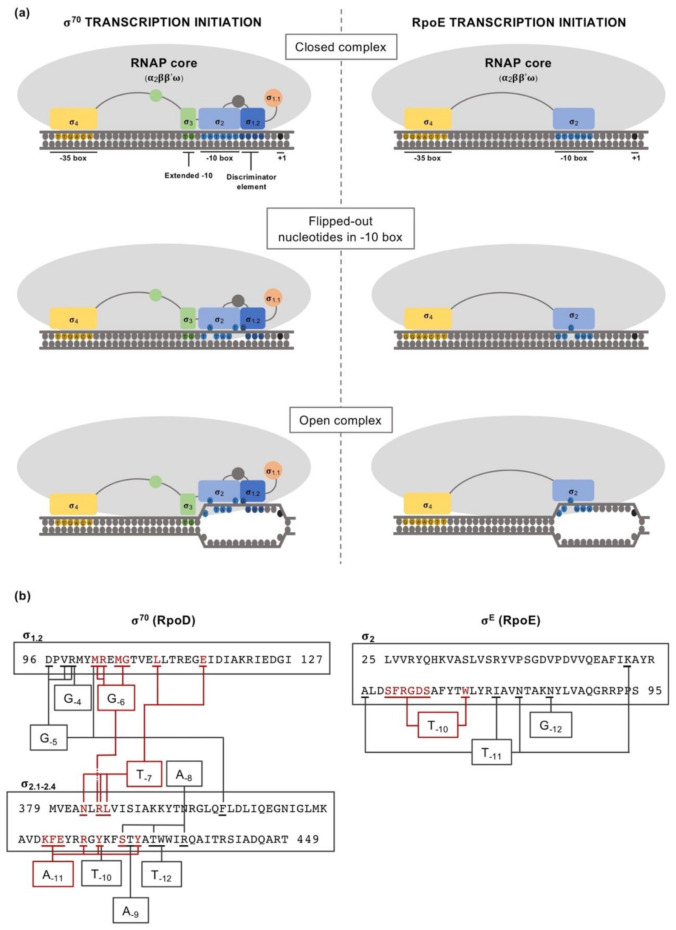
Comparison between the transcription initiation mechanisms of the primary σ^70^ factor (RpoD) and the extracytoplasmic function σ factor (ECF) RpoE, from *E. coli*. (**a**) Involvement of the different σ factor domains (using the same color code as in Figure 1) in the transcription initiation, emphasizing the differences between the vegetative σ^70^ factor and RpoE regarding their promoter melting capability. The different isomerization stages from closed complex to open complex are indicated. (**b**) Schematic of the contacts between -10 box nucleotides and σ_2_ domain residues for RpoD and RpoE. Residues directly interacting with -10 box nucleotides are underlined. Nucleotides that are flipped-out during transcription initiation and the respective residues that contact them are indicated in red.

Taken as a paradigmatic example, *E. coli* σ^70^ factor (RpoD) is composed of several domains and subdomains [15,17,38,39] each of them with a specific role, namely: σ_1.1_, σ_2 (1.2, NCR, 2.1, 2.2, 2.3 2.4)_, σ_3 (3.0, 3.1)_, σ_3.2_ and σ_4 (4.1, 4.2)_ (Figure 1). Once the RNAP holoenzyme has formed, domains σ_1.2_, σ_2_, σ_3.1_ and σ_4_ of the σ^70^ are exposed on the surface of the holoenzyme and provide the promoter recognition and binding activity (Figure 2a). Upon promoter binding, domain σ_2_ triggers the separation of the dsDNA at the conserved -10 motif of the promoter to form a transcription bubble or open complex [23]. Separation of the double strand is further augmented by the region σ_1.2_ [40,41], considered to be an extended part of the σ_2_ region [17], which melts the promoter at the discriminator element (details on the promoter melting capacity of the σ^70^ factor compared to ECFs are further explained at the end of this section). The σ_NCR_ (non-conserved region) subdomain is an adjacent region to the core σ_2_ domain whose interaction with the core RNAP, in a manner which is antagonistic to the core RNAP–σ_2_ interaction, facilitates promoter escape allowing the first stages of elongation to occur [42]. σ_3.2_ is a conserved linker between the σ_3_ and σ_4_ regions that inserts into the RNAP active site and occupies the RNA exit channel. Part of this region, the σ finger, interacts with the DNA template strand pre-organizing it into a helical conformation for accommodating it into the RNAP active site, thus enabling the binding of initiating NTPs to the template strand. For promoter escape and transcription elongation to occur, the DNA–σ_3.2_ interactions must be disrupted and the σ finger has to be displaced from the RNA exit channel, which may explain from a mechanistic perspective the close relationship between this region and abortive transcription events [23,29,43,44].

As mentioned above, Group IV σ factors consist of only the σ_2_ and σ_4_ DNA-binding domains and a non-conserved σ_2_–σ_4_ linker of less than 50 residues, which is highly divergent in length and sequence among ECFs. Most of the knowledge about the mechanisms of transcription initiation by ECFs comes from the well-studied *E. coli* RpoE (σ^E^). Crystal structures of RpoE σ_4_ complexed with the −35 element [45], σ_2_ complexed with the −10 element [46] and the structure of the transcription initiation complex formed by the RpoE–RNAP holoenzyme [35,36,37] have identified protein–DNA interactions critical for promoter recognition and DNA melting, and protein–protein interactions essential for the assembly of the RpoE–RNAP holoenzyme.

Apart from the differences in the interaction with the -10 box between ECFs and primary σ^70^ factors, divergent mechanisms for the recognition of the conserved -35 sequence motif have also been described [45,47]. In Group I σ factors, abundant nucleotide-specific interactions are stablished between the σ_4_ domain and the -35 box, for example, using the *Thermus aquaticus* σ^70^ factor as a model for structural studies [45]. When determining the interaction between the σ_4_ domain of the ECFs RpoE (*E. coli*) and SigW (*B. subtilis*), with target -35 boxes GGAACTT and ATTGAAACCTTT, respectively, numerous additional interactions also occurred, except with the central short A-tract [45,47]. Interactions with the A-tract happened to be fewer and weaker, which contrasts with the widespread conservation of the central A-tract in the target -35 box of most ECFs. The explanation for this, which is suggested to apply for other ECFs, is that the presence of this A-tract provides the appropriate DNA geometry within the -35 box for its interaction with the σ_4_ domain.

The recent structural studies of transcription initiation complexes using the *Mycobacterium tuberculosis* σ^H^, σ^L^ and chimeric σ^H^/^E^ factors revealed that the σ_2_-σ_4_ linker, similarly to the aforementioned σ finger in the primary σ^70^ factor [23], inserts into the active site of the core RNAP, interacting with the template single-stranded DNA, and occupies the RNA exit channel [35,36,37]. Besides, this similarity with the σ_3.2_ region of the σ^70^ factor is also suggested by analysis of mutant versions of this region in different ECFs, which show, to different extents, an inability to interact with the core RNAP and isomerize the holoenzyme–DNA closed complex to an open complex. This also affects transcription initiation and the production of abortive transcription complexes (thus indicating a role in promoter escape). However, unlike the σ_3.2_ region of the primary σ^70^ factor, the amino acid sequence of the σ_2_–σ_4_ linker is not conserved. Rather, ECFs share a similar secondary structure at the ends of this linker, which may indicate, together with the results obtained from structural studies, that these regions of the linker are important for its activity within the RNAP active site and the exit channel [35,36,37].

While the primary σ^70^ factor drives the majority of bacterial transcription, alternative σ factors, including ECFs, target small groups of operons. This implies that σ^70^ must have a broader promoter specificity, whereas alternative σ factors must stringently promote the transcription of their regulon alone [46,48]. The key to this difference resides in their respective mechanisms of promoter recognition and melting of the dsDNA at the -10 box. Primary σ^70^ factors melt the promoter mainly at the AT-rich -10 sequence of the non-template strand through a set of invariant aromatic residues involving regions σ_2.3_–σ_2.4_, opening promoters by flipping out two bases, A_-11_ and T_-7_, at the -10 box in *E. coli* [17]. Additionally, upon interaction with the discriminator element, the region σ_1.2_ is also able to flip out the G_-6_, thus increasing the melting capacity of the primary σ^70^ factor [23,40]. The amino acid residues within the σ_2_ domain that interact with each of the nucleotides in the -10 box and the discriminator element are detailed in Figure 2b, according to [23,40]. The ability of the σ^70^ holoenzyme to induce the DNA melting from three different positions allows a certain divergence in these nucleotides with respect to the consensus A_-11_, T_-7_ and G_-6_. This ensures that, even if a mismatch in any of those positions has a lower potential to be unstacked and buried by the σ^70^ factor, the open complex can still be formed thanks to the ability to flip out the other nucleotides. A distinctive feature of Group IV σ factors is that the regions σ_2.3_–σ_2.4_ are highly divergent from primary σ factors, with region σ_2.3_ lacking crucial melting residues [46]. Promoter melting by ECF σ factors involves an adjacent variable protein loop region within domain σ_2.3_ (loop L3), which is essential for specific recognition of only one flipped-out nucleotide at the −10 element (specifically, the -10 nucleotide). This is due to the presence of a pocket in which the flipped-out nucleotide is accommodated, where specific interactions are stablished with all the functional groups of the respective nucleotide [46,49]. In terms of the melting capability, this explains that any divergence in the -10 position from the consensus target promoter of a given ECF drastically reduces, if not abolishes, transcription [46]. For example, *E. coli* RpoE requires a cytosine in the -10 position (as for the σ^70^ factor, the detail of the residues within the σ_2_ domain of RpoE that specifically interact with each of the -10 box nucleotides is shown in Figure 2b, according to [46]). Swapping this “specificity loop L3” of *E. coli* RpoE for the respective loops of *Bacillus subtilis* σ^W^, σ^X^ or *Sphingomonas melonis* EcfG redirected the specificity of the RNAP holoenzyme to the target sequence of those σ factors, which only differ in the -10 nucleotides. Altogether, this explains the reduced melting capacity and high promoter stringency of ECFs, which prevents non-specific transcription initiation, in contrast to primary σ^70^ factors, characterized by a high promoter-melting capacity required to drive the bulk of bacterial transcription from promoters where A_-11_ and T_-7_, and additionally G_-6_, are conserved [46,49].

These specific ECF–promoter interactions at the -35 and -10 boxes have been recently explored to implement a tool for promoter prediction based on the amino acid composition of key DNA contact points within the σ_2_ and σ_4_ domains [50]. Its application has allowed the prediction of target regulons of nearly 70% of the known ECFs and established that around 55% of them are autoregulated.

## 4. ECF Classification

Although ECFs show sufficient sequence and structural similarity to be clustered as homologs in Group IV, their diversity is sufficient to allow their classification into distinct sub-groups based on functional similarities, and this facilitates their further study. The first attempts at their formal classification was published in 2009 by Anna Staroń and collaborators [12]. At that time, 369 available bacterial genomes were screened for ECF sequences, and up to 2708 non-redundant ECFs were predicted according to their sequence similarity, phylogenetic analysis and genomic context conservation. As a result, a total of 67 sub-groups were established, with 43 defined as “major” (ECF01-43) and “minor” (ECF101-124, represented by less than 10 protein sequences), whilst 835 ECFs remained unclassified. The joint publication of the tool *ECFfinder* together with the classification helped to describe new ECFs, and to predict their promoter, their input stimuli, their regulatory mechanism and possible target genes just by analyzing their primary sequence. However, a major flaw in this classification was the reduced number of bacterial genomes available at that time and a considerable bias toward highly represented phyla (86.9% of the genomes belonged to Proteobacteria, *Actinobacteria* and *Firmicutes*). Later on, with the analysis of nine newly sequenced *Planctomycetes* genomes [51] and more than 150 actinobacterial genomes identified [52], the classification was expanded to 94 groups [53]. At this point, the distinction between “major” and “minor” groups was abandoned, as it proved to be arbitrary due to an effect of the number of analyzed genomes and ECF representatives. Additionally, based on this small subset of studied ECFs, some hallmark features of these proteins could be established: (i) they recognize promoters with AAC as -35 box and CGT as -10 box; (ii) they are formed by only the σ_2_ and σ_4_ domains separated by a linker of less than 50 residues; (iii) they are co-transcribed with a cognate anti-σ factor; and iv) they are positively autoregulated (except for a few examples, such as the FecI-like ECFs) [12]. After the findings revealed by the subsequent updated versions of the ECF classification, the defining features of an ECF were restricted to presenting only the σ_2_ and σ_4_ domains as promoter-binding determinants and key contact regions with the core RNAP. These contacts are mainly those of the surface between the σ_2.2_ region and the β’ subunit, but also those of the surface between the σ_4_ domain and the β subunit with a weaker intensity [34,54,55]. Beyond that, their increasing diversity regarding their regulatory mechanisms, their target promoters, the signals to which they respond and which they regulate, or their genomic context, do not allow us to set any further clear-cut rules for defining an ECF as such [53].

The advent of bacterial genome sequencing and annotation projects has led to a great increase in the number of available sequences in the databases, which has helped mitigate the former bias toward highly represented phyla. In its latest version [56], the classification has undergone an important reorganization and expansion, increasing its accuracy, mainly due to the increase in the number of analyzed genomes, and organizing the ECFs into monophyletic groups within the ECF phylogenetic group. At the time when the analysis was performed (2017), more than 180,000 bacterial genomes were available at the National Center for Biotechnology Information (NCBI) database and were used as an initial dataset. Based on this analysis, a library of more than 170,000 non-redundant ECF sequences could be obtained (around a 50-fold increase), and after the study of their sequence similarity and phylogeny and genomic context conservation, a total of 157 distinct ECF groups were defined. Among them, 62 of the original groups remained and were expanded, while 22 new groups were defined. Additionally, other sub-groups that were originally formed based on limited similarity among their members disappeared, and their members were redistributed among other sub-groups in which they fitted better, or instead, if very divergent, they were classified as ungrouped. This may be due to the lack of similarity with any of the established groups, so that they cannot be included, and the low number of representatives sharing sequence similarity and synteny, not reaching the minimal threshold to form a dedicated group. Altogether, this improvement in the classification will generally improve the ability of the scientific community to predict the function of newly annotated ECFs. Thus, it will help to: (i) define their target promoters based on over-represented motifs in those that are autoregulated, (ii) determine functionally important residues for this recognition and (iii) develop predictive tools to define target promoters, especially for those ECFs that are not autoregulated (a matter that has already started to be addressed [50]). Furthermore, a continuous update is guaranteed with the constantly increasing number of bacterial genome sequences.

**Figure 3:**
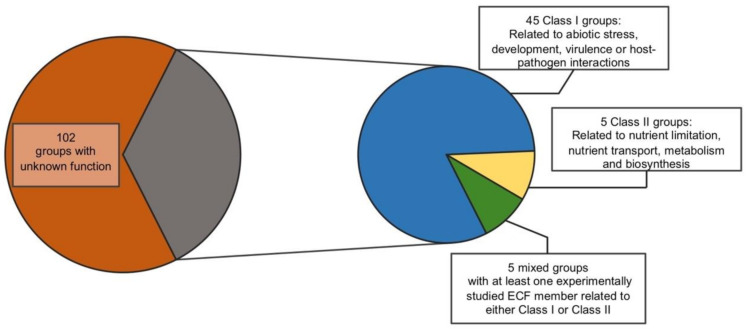
Diagram of the different groups of ECF σ factors according to their possible functional role. Note that most of the groups of ECFs do not have a single experimentally characterized representative.

Despite the classification being based on sequence similarity and genomic context of ECFs, nevertheless, due to their degree of conservation, target promoter motifs and potential functions can be inferred for each distinct group with a high level of accuracy based on the few experimentally studied representatives. Even in groups with no experimentally analyzed representatives, regulatory mechanisms and target motifs may be predicted based on the genomic context and the amino acid composition of the DNA-binding domains compared to other ECFs [56]. Regarding their function, although ECFs are not classified based on it, their conservation also allows inferences to be made regarding the physiological processes in which they are involved, again based on the studies of a number of representatives. However, ascribing a specific exclusive function to some of the ECF groups might be risky due to the small amount of manually studied examples within them. For instance, some members of the ECF41 are known to be involved in the protection against oxidative stress [57], but other representatives of this group do not participate in that response, or any other known so far [58]. Reviewing the latest ECF classification [56] and based on the manually studied examples of each group (which are also provided in the classification), we have attempted to cluster them function-wise into three broadly related classes: Class (I) are ECFs involved in responding to external or internal cues that may be directly deleterious to maintaining cell integrity and homeostasis. These include responses such as resistance to abiotic stress, to development, virulence and interaction with host organisms; Class (II) are ECFs involved in responding to metabolic and nutritional cues, including nutrient limitation, transport and biosynthesis; and Class (III), in which there are groups including those of unknown function. This is mainly due to the lack of studied ECFs within these groups (depicted in Figure 3; ECF groups within each class are listed in Supplementary Table S1). We have found that 45 ECF groups of class I, five groups of class II and five groups could be classified in both of the aforementioned functional clusters, given that several of the studied examples have functions that are related to processes that could be ascribed to both classes. It is also worth mentioning that, thanks to this functional screening, 102 ECF groups are noted as unstudied, which reflects the high diversity of ECFs and which are yet to be explored. Since the majority of the members of each ECF group remain unstudied, it is possible that some of their specific functions may not fit precisely with the broad functions assigned in this classification. However, it gives an idea of the importance of ECFs for stress responses and may serve as a starting point to tackle the characterization of newly studied ECFs in combination with the additional information provided in the ECF classification [56].

## 5. Regulation of the ECF Activity and Production

**Figure 4:**
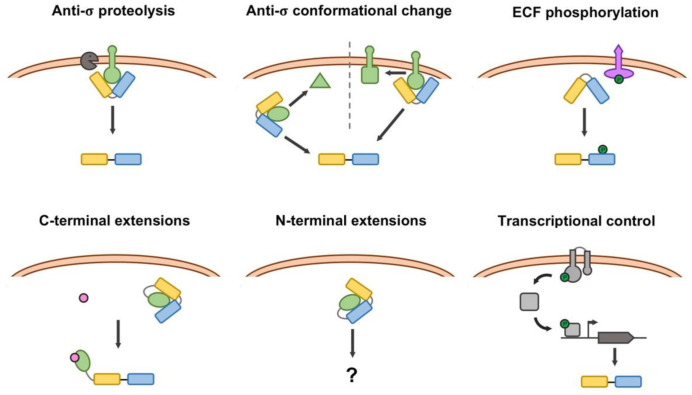
Representation of the different modes of control of ECF σ factor function. Blue and yellow boxes represent σ_2_ and σ_4_ domains of ECFs, respectively; anti-σ factor and regulatory extensions are represented in green; proteases appear in black; systems controlling the expression of the target ECF, such as two-component systems (TSCs), are depicted in gray; Ser/Thr protein kinases appear in magenta; phosphoryl groups are depicted as green circles, and inducer ligands appear as pink circles.

From the perspective of the regulation of their activity, the functional control of ECFs can be grouped into five general mechanisms, although each type of ECF may present distinctive regulatory features [59]. These include regulation by membrane-anchored anti-σ factors, by soluble anti-σ factors, through serine/threonine protein kinases (STPKs) and via C- and N-terminal extensions. Additionally, some ECFs may be regulated exclusively by controlling their production at the transcriptional level. All these regulatory mechanisms are represented in Figure 4. Of these, membrane-anchored anti-σ factors represent the ancestral regulatory mechanism for ECFs from which the rest of them emerged [59], and is the most widespread (74% of the ECFs with a known regulatory mechanism). Soluble anti-σ factors arose from the ancestor membrane-anchored proteins by means of gene truncations that led to a separation of the cytosolic anti-σ domain from the transmembrane helices [59].

Anti-σ factors are usually encoded by genes located in the vicinity of the gene encoding the target ECF, or even within the same transcriptional unit. They prevent the ECF from functioning by titration, while it is not needed. To release ECFs from inhibition, anti-σ factors may undergo proteolysis, the most frequent mechanism for membrane-bound anti-σ factors [59], or they may undergo conformational changes (or partner-switching events in the case of examples related to the regulation of the alphaproteobacterial general stress response, explained in detail below) [22]. Crystallography and homology modelling studies have revealed the molecular aspects of this ECF-anti-σ binding in different pairs. Although the molecular details of the contact surfaces may differ among pairs, the global picture shows that anti-σ factors occlude the contact regions of the cognate ECF required for the interaction with the RNAP and lock the ECF in a non-optimal conformation that prevents binding of the target promoter [60]. This has been discussed as a universal ECF mechanism to prevent the formation of trimeric complexes between the ECF-anti-σ pair and the RNAP or the target DNA sequence [61]. According to their secondary structure, which is indicative of their mode of interaction with their ECF pair, anti-σ factors can be classified in three groups, depending on the presence of a class I, class II or class III anti-σ domain (respectively, ASDI, ASDII or ASDIII). In the RpoE-RseA and SigW-RsiW ECF-anti-σ pairs from *E. coli* and *B. subtilis*, respectively [62,63], which are representatives of class ASDIs, the anti-σ factor locks the ECF in a conformation that is unable to bind either the RNAP or the target DNA sequences. Recent computational and phylogenetic analyses show a coevolution between the contact surfaces of ASDIs and their respective ECF pair [59].

Within ASDIIs, structural studies have reported that in the EcfG-NepR and CnrH-CnrY pairs from *S. melonis* and *Cupriavidus metallidurans*, respectively [22,64], the anti-σ factor embraces the outer surface of the ECF, preventing the interaction with the RNAP. Finally, a structural study of the ECF-anti-σ interaction of the BldN-RsbN pair from *Streptomyces venezuelae* [60] shows that RsbN is able to prevent BldN from binding the RNAP, by directly blocking access of the σ_4_ domain to the -35 box and by sequestering BldN to the membrane, as the anti-σ is a membrane-anchored protein. By comparing the structure of ASDIs and ASDIIs, RsbN served to define the ASDIII anti-σ factor class for the first time.

STPKs were initially thought to have a minor role in signal transduction in *Bacteria* compared to their major regulatory roles in *Eukarya*. However, the presence of coding genes for STPKs in bacterial genomes revealed through the numerous genome annotation projects developed in recent years has attracted the attention of microbiologists. In particular, ECFs classified into groups ECF43, ECF59 and ECF60 (the latter no longer appearing in the latest classification, with its original members not clustering in any of the current ones [56]) are encoded by genes located near genes encoding an STPK. Further analyses of this synteny in *Xanthomonas citri* revealed that a neighboring STPK (PknS) is essential for the activation of its cognate ECF (EcfK), which regulates the Type VI Secretion System of this strain [65]. Recent studies have shed light on the mechanistic aspects of this regulation in the pair formed by EcfP and the STPK PknT in *Vibrio parahaemolyticus* [66], which controls polymyxin resistance. One key contact between the negatively charged DAED motif located in the σ_2_ domain of σ factors and the positively charged helices located in the β’-clamp of the core RNAP is essential for the formation of the holoenzyme [66,67]. In STPK-regulated ECFs, the DAED motif is substituted by non-charged residues (STTA in EcfP). Upon phosphorylation of a threonine (ECF43) or serine (ECF59 and the original ECF60) residue, the negative charge is restored, thus enabling the interaction of the ECF with the core RNAP [66].

As mentioned above, ECFs have been defined by the presence of domains σ_2_ and σ_4_ alone, which together minimally define a σ factor. The gene encoding some of these ECFs does not appear near any gene encoding a putative anti-σ factor or accessory regulatory protein that might control function. Instead, they exhibit extensions in their open reading frames, mainly at their C-terminus, but some also have an N-terminal extension (reviewed in detail in [14]). These extensions are involved in regulation of function of the cognate ECF. In recent studies, it has been suggested that these ECFs may have originated by gene fusion events at different points throughout evolution, possibly with sequences encoding anti-σ factor-like proteins [14,59]. These elements have been detected in up to 18 phylogenetically distinct groups of ECFs and in the few cases studied experimentally, the extensions seem to act as ligand-binding domains that maintain the ECF in an inactive conformation when the ligand is absent. According to the original classification, the experimentally studied groups of ECFs with C-terminal regulatory extensions were clustered into three groups: (i) ECF41 σ factors, including SigJ and its SnoaL_2 domain, from *M. tuberculosis* [68]; (ii) the members of the ECF42 group, containing a tetratricopeptide repeat (TPR) domain, described in *S. venezuelae* and *Xanthomonas campestris* [69,70]; and (iii) the metal-responsive ECFs that include the current ECF238 group (which includes the ECF44 group) [56], in particular CorE and CorE2 from *Myxococcus xanthus*, which are the only experimentally studied members [71]. However, through the analysis that led to the latest classification, many C-terminal extension-containing ECFs appear to be distributed within the different groups, so they might not be restricted to the aforementioned three groups [56].

Finally, ECFs with N-terminal extensions have been recently discovered after the analysis of more than one hundred *Planctomycetes* genomes [72] and classified within the original ECF87, ECF88 and ECF89 groups. Although none of the members of this group has been experimentally studied, the position of the extension (at the front of the holoenzyme when it moves over the DNA template) implies novel aspects in the interaction of these ECFs with the core RNAP and suggests a different regulatory mechanism that is yet to be understood [14]. In the first instance, ECFs with this structural feature were classified in the original ECF87, ECF88 and ECF89 groups. However, after the latest phylogenetic analysis that resulted in the current classification, these groups no longer exist, and ECFs containing N-terminal extensions appear scattered in a number of groups forming specific subgroups within them [56].

In the case of orphan ECFs, with no regulatory partner associated (either anti-σ factor or regulatory extension), the control of their transcription, frequently by TCSs, may stand as the only regulatory mechanism controlling their function [59]. Such is the case for the virulence regulatory genes of the corn pathogen *Pantoea stewartii* subsp. *stewartia* [73]. In this example, the HrpXY TCS activates the transcription of the RpoN-dependent transcriptional activator *hrpS*. This regulator is able to fully activate the transcription of the ECF HrpL coding gene, which eventually drives the transcription of the *hrp* and *wts* virulence factors.

Expression of most of the ECF-containing operons is induced in response to a specific signal. In most instances, with a few exceptions such as the FeclR-like systems and some others [12], they are autoregulated by a positive feedback loop dependent on the regulated ECF itself. An intermediate situation of this type of transcriptional regulation is found in the general stress response (GSR, a global response mediated by ECFs in Alphaproteobacteria that is discussed in detail below) regulatory system described for *Sphingopyxis granuli* TFA [74]. Here, upon activation of the GSR signaling pathway, the constitutively expressed ECF EcfG2 drives the transcription of the paralogous EcfG1, which is also able to control its own transcription. This contrasts with the canonical GSR regulatory mechanism (further discussed below), where one EcfG representative is able to autonomously regulate its expression under GSR-inducing conditions.

In spite of these basic regulatory mechanisms that govern the activity of most of the ECFs, there are still cases in which a possible regulatory mode is not clear or cannot be easily predicted. Traditionally, the regulatory partner of an ECF has been its cognate anti-σ factor, which is usually encoded in the same locus. This is also true for STPK- and many TCS-regulated ECFs, according to analysis of conservation of the genomic context used for their classification. However, some ECFs are encoded by a monocistronic operon with no context conservation, or if the genomic context is conserved, unknown elements or elements from which a possible regulatory activity cannot be inferred easily, are found in the vicinity. In the case of ECFs with regulatory extensions, even if they are encoded by isolated genes, a regulatory mechanism can still be predicted. Nevertheless, questions remain about the regulation of ECFs encoded without an obvious cognate anti-σ factor or another putative regulator nearby. There is the possibility that a cognate anti-σ factor actually exists, although it would have to be encoded elsewhere in the genome. For instance, in *Sinorhizobium meliloti*, the ECF RpoE2 is regulated by the anti-σ factors RsiA1 and RsiA2, with RsiA2 being encoded in the same operon as RpoE2 and RsiA1 being encoded at a different position on the chromosome [75]. Furthermore, in *Rhizobium etli*, there are two EcfG σ factors that control its general stress response, with *ecfG1* located on the chromosome and *ecfG2* located on a plasmid [76]. However, the only putative NepR anti-σ factor (the typical regulator of EcfG σ factors) identified so far is encoded on the chromosome. Another issue in the search for putative anti-σ factors is the difficulty regarding their annotation. Anti-σ factors share a poorly conserved sequence similarity, and although their secondary structure presents a more conserved pattern for those belonging to the same anti-σ class [12,77,78], the bioinformatic identification methods still rely on previously identified anti-σ proteins. This, along with the presence of unknown elements encoded in the vicinity of many ECFs [56], may represent a negative bias in the identification of elements regulating the activity and/or production of these σ factors, limiting the findings of these searches to elements sharing similarity to those that have been experimentally studied beforehand.

## 6. Examples of Model ECFs: RpoE and EcfG

Since their discovery, a wide variety of ECFs has been experimentally studied without even knowing that they might be evolutionarily related. In this section, we will summarize the main characteristics of two important examples of ECFs involved in stress responses, and whose mechanisms regulating their function are complex and differ dramatically. Firstly, we will focus on the ECF02-type RpoE, one of the first representatives of these proteins to be discovered that is regulated by regulated proteolysis of its membrane-anchored anti-σ factor RseA. We will mainly discuss the role of the *E. coli* homolog of RpoE, since it has been profoundly characterized at the molecular level with regard to its response to cell-envelope stress. Additionally, we will dedicate a section to the ECF15-type EcfG, the master regulator of the GSR in Alphaproteobacteria, an ECF that activates a global response whose activity is regulated by a unique partner-switching mechanism.

### 6.1. RpoE Stress Response

RpoE was initially identified in *E. coli* due to its ability to initiate transcription from the third promoter of *rpoH* (*rpoH* P3) at lethal temperatures (50 °C) [79]. Transcription from this promoter allows the synthesis of another alternative σ factor, the heat-inducible σ^H^ factor, responsible for the transcription of heat-shock response genes, encoding products consisting primarily of chaperones and proteases that bind to cytoplasmic proteins and assist them in folding. Apart from *rpoH*, the second gene known to be transcribed by RpoE was *htrA* (high temperature requirement, also called *degP*). *htrA* encodes a 51 kDa precursor protein that is processed to the 48 kDa periplasmic endopeptidase DegP, required for growth and survival at high temperatures (>42 °C) [79,80]. At that time, many efforts were focused on identifying the signals that modulate RpoE activity, and the periplasmic localization of DegP was a first indication that the RpoE regulon might be involved in protecting the extracytoplasmic compartment from stress [79]. In 1993, Mecsas and collaborators [81] found that RpoE activity increased when outer membrane proteins (OMPs) where over-produced and decreased when they were under-produced, suggesting that the inducing signal generated by OMPs originated in the periplasm or at the outer membrane (OM). In fact, the presence of unfolded OMPs in the periplasm is the most thoroughly characterized signal for RpoE activation. However, defects affecting lipopolysaccharide (LPS) biosynthesis and assembly also function to elicit the signal of the RpoE envelope stress response. All these findings indicate that robust signal transduction in response to periplasmic stress requires these dual molecular signals, rather than either signal on its own [82,83].

In general, any kind of environmental stress that causes accumulation of misfolded proteins in the periplasm, such as heat-shock, the presence of ethanol, pH changes, oxidative stress or osmotic stress, results in the activation of the RpoE pathway. In addition, RpoE activity is induced by entry into stationary phase [84] and participates in biofilm formation and resistance to antimicrobial agents [85,86]. Essentially, RpoE integrates different signals, all of them related to dysfunction in outer membrane biogenesis, and triggers a protective response activating damage repair pathways. This adaptive response involves the expression of genes related to the biogenesis, transport, and/or assembly of LPS, phospholipids, OMPs and proteases and chaperones that maintain or repair OM integrity [87]. In addition to maintaining the cell envelope integrity under stress conditions, RpoE is essential during normal growth in *E. coli*, in contrast to other ECFs.

Part of its regulon also includes regulatory small RNAs (sRNAs), such as MicA and RybB, through which RpoE downregulates all major OMP synthesis pathways and controls the remodeling of LPS composition [88]. Moreover, another RpoE-dependent sRNA is MicL (SlrA [89]), which downregulates the synthesis of the envelope murein lipoprotein Lpp until stress is relieved. They constitute the post-transcriptional repression arm (sRNA arm) of the RpoE response, which, together with the transcriptional activation arm (protein arm), maintains the correct balance of all the components of the cell envelope and preserves the cell envelope homeostasis [90]. MicA and RybB have strong RpoE-dependent promoters and are considered global regulators, since they target more than 30 mRNAs in *E. coli.* Their targets not only include major porins, resulting in inhibition of synthesis of all major OMPs (in fact, porins are 1/3 of the targets of these sRNAs), but also non-OMP targets without envelope-related functions are regulated. MicA and RybB also converge in some messenger RNA (mRNA) targets such as *lamB*, *ompA*, *ompW* and *tsx* [90]. They seem to function by relieving stress due to imbalance in the OM when the RpoE activity is inadequate, since they can prevent cell death associated to deficient RpoE activity [90]. MicA includes among its non-porin targets the *phoP* mRNA. The PhoPQ TCS regulates genes involved in Mg^2+^ transport, resistance to antimicrobial peptides, acid pH resistance, virulence and also LPS modification under certain stress conditions. Therefore, MicA links these cellular processes to RpoE [91,92]. Similarly, RybB is involved in the translational repression of the glycosyltransferase WaaR, resulting in the alteration of the global LPS composition [88]. The *micL* (*slrA* [89]) transcript originates from the 3′ untranslated region of a gene named *cutC*. *micL*, like *micA* and *rybB*, is transcribed from a strong RpoE-dependent promoter located within the coding sequence of this gene, and the transcript is further processed into a smaller transcript (MicL-S). MicL represses the translation of Lpp, an OM lipoprotein that forms covalent linkages between the OM and the peptidoglycan cell wall and is the most abundant protein produced by *E. coli*. However, Lpp has no role in OMP or LPS biogenesis itself, so it has been proposed that reducing the Lpp levels could be a way to help the biogenesis machinery, such as the Lol pathway, to deliver other lipoproteins that do have essential functions in OMP and LPS biosynthesis [93]. Nevertheless, further research is required to determine why reducing Lpp levels during RpoE-inducing stress conditions is necessary. In addition, new interactions of MicL with other RNA partners have been identified recently by RIL-seq, which would extend its potential targetome and, thus, indirectly the RpoE-dependent regulation [94].

Two promoters were initially reported to direct the *rpoE* transcription *(rpoE* P1 and *rpoE* P2), being *rpoE* P1 a distal promoter region of unknown regulation and *rpoE* P2 positively regulated by RpoE itself in response to OMPs misfolding and LPS defects [80]. Further analyses aimed at studying transcriptional regulation of *rpoE* have clarified the whole *rpoE* promoter organization and identified a total of six promoters (*rpoE* P1–P6, from distal to proximal, with transcription start sites located in positions -381, -327 (shared for P2 and P3), -218, -153 and -75, respectively). This organization sustains the *rpoE* transcription in response to different signals by recruiting different σ factors, TCS response regulators and global regulators that function on individual promoters [95]. Among those six promoters, the current *rpoE* P6 would correspond to the originally named *rpoE* P2, and the current *rpoE* P4 would approximately correspond to the original P1. This complex organization and regulation places RpoE as a pivotal regulatory element, where a variety of stresses may converge allowing a rapid response.

Briefly, according to [95], the *rpoE* P2 promoter shares the same transcription start site as *rpoE* P3 and is regulated by RpoN using QseF as an enhancer-binding protein. QseF is a response regulator of the QseEF TCS with homology to the NtrC family of transcriptional activators. The RpoD-dependent *rpoE* P3 promoter is noteworthy, as it is able to respond to specific LPS defects related to LPS biosynthesis, and this induction is dependent on the Rcs signaling pathway. The RcsBCDF phosphorelay senses damage at the LPS layer particularly in the RcsF OM lipoprotein and transmits it to the RcsB cytoplasmic response regulator, which then binds *rpoE* P3. The *rpoE* P2 promoter also responds to LPS defects but to a much lower extent than the *rpoE* P3 promoter. The *rpoE* P4 promoter behaves in vivo as a typical RpoS-dependent promoter, in the sense that the *rpoE* P4 promoter could be involved in inducing the RpoE pathway under stationary phase and general stress conditions [95]. The autoregulated promoter *rpoE* P6 responds to availability of free RpoE in a positive feedback loop as a result of its transcriptional and post-transcriptional activation (explained below).

**Figure 5:**
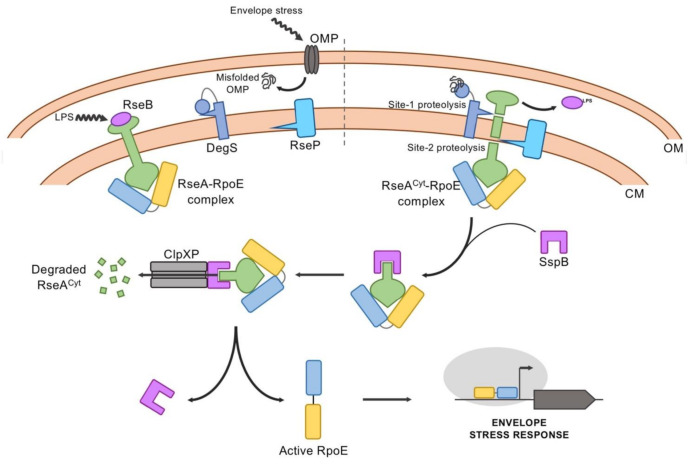
Regulatory model of the RpoE-mediated envelope stress response. In the absence of stress, RpoE remains sequestered by the membrane-anchored anti-σ factor RseA, which binds the RseB protein in its periplasmic domain. Additionally, the PDZ domain in the protease DegS blocks its own active site. As a first step in the stress response, DegS is able to interact with the C-terminal domain of misfolded OMPs, and off-pathway LPS accumulated in the periplasm relieves RseA from RseB binding. Then, two subsequent proteolytic cleavage events in sites 1 and 2 by proteases DegS and RseP, respectively, release the cytosolic domain of RseA (RseA^Cyt^) bound to RpoE to the cytosol. Here, the complex is bound by the adaptor protein SspB that leads it to the major protease ClpXP. This protease degrades the remaining domain of RseA, thus releasing RpoE to drive the expression of its target genes.

The factors that regulate the activity of RpoE include the RseA anti-σ factor and RseB as negative regulators, and DegS and RseP, two inner-membrane proteases that together with the cytoplasmic proteases (the ClpXP complex), sequentially inactivate RseA by regulated intramembrane proteolysis (RIP). The circuit generated by these proteins provides information on the integrity/health of the cell envelope, ultimately determining RpoE activity, and many of the molecular details have been described [83,96] (Figure 5).

RseA is an integral membrane protein and its N-terminal cytoplasmic domain functions as an anti-σ, titrating RpoE, keeping it in an inactive conformation. The co-crystal structure of this cytoplasmic domain complexed to RpoE has revealed that RseA inhibits RpoE activity by sterically blocking its association to the RNA polymerase, as explained above. Biochemical studies have shown that RpoE has higher affinity for the RseA cytoplasmic domain than to the core RNAP [62]. Only the proteolytic degradation of RseA is able to release RpoE from inhibition, giving the core RNA polymerase the chance to interact with it, so that the RNAP holoenzyme can activate gene expression to initiate the stress response. Therefore, proteolytic degradation of RseA under stress conditions is tightly regulated.

DegS (site-1 protease), as a member of the HtrA family, harbors a catalytic serine protease domain and a periplasmic C-terminal PDZ domain. PDZ domains are protein modules that mediate specific protein–protein interactions and bind preferentially to the C-terminal 3–4 residues of the target protein. They are found in signaling proteins of *Bacteria* and *Eukarya*, which also negatively regulate their protease activity [97]. The periplasmic PDZ domain of DegS also acts as a sensor of cell envelope stress. The current model proposes that the C-terminal PDZ domain negatively regulates DegS protease activity, and that this inhibition is relieved when the PDZ domain binds to C-terminal peptides that are provided by unfolded, unassembled or denatured OMPs during environmental stress. These sequences are kept inaccessible in properly folded OMPs, but they are exposed and accumulate in the periplasm if OMP folding is defective. This peptide binding reorients the active site of DegS to facilitate catalysis, triggering the proteolytic cascade that degrades RseA [98,99]. RseB, a periplasmic protein encoded in the *rpoE* operon, binds to the periplasmic domain of RseA. RseA cleavage by DegS requires binding disruption of RseB [82]. The RseB–RseA dissociation is proposed to be caused by LPS binding, although it is still an open question how lipophilic free LPS could accumulate in the periplasm, and if it can do so, the nature of the RseB signaling that allows DegS-dependent site-1 cleavage of RseA [82]. Therefore, as biogenesis of OMP and LPS appear to cross-talk with each other, dysfunction of either of them can initiate RseA proteolysis [83]. The subsequent site-2 cleavage of the resulting intermediate of RseA occurs within the inner membrane and is carried out by RseP. This is an inner-membrane zinc-metalloprotease with four transmembrane helices and two PDZ domains that control its proteolytic activity. Once the cytoplasmic domain of RseA in complex with RpoE is released into the cytoplasm, it binds to the SspB adaptor protein, which delivers the complex to the cytoplasmic protease ClpXP. This proteolytic complex degrades the RseA fragment, thus allowing the released RpoE to bind to the core RNAP [100].

It has been argued that phosphorylation of RseA could be another mechanism regulating its interaction with RpoE [101]. In that sense, the Tyr kinase Etk, in concert with the antagonistic Tyr phosphatase Etp, was found to phosphorylate RseA. Phosphorylation of the Tyr36 residue of RseA, located at its N-terminal domain, which is responsible for binding RpoE, could alter its binding affinity by this σ factor, and thus, the activation of its regulon.

Another layer of control over the expression of RpoE is mediated by the alarmone guanosine 3,5-bispyrophosphate (ppGpp) and DksA under nutrient limitation [102]. This entails an RseA-independent regulation of the RpoE activity. ppGpp is well known as a general signal of starvation stress (stringent response) and its cellular concentration fluctuates due to changes in nutritional conditions. On the other hand, DksA is a 151 amino acid zinc-containing protein that binds to RNA polymerase (RNAP) affecting different aspects of open complex formation and transcription initiation [103]. Measuring the activity of RpoE by using some RpoE-dependent promoters, such as the *rpoH* P3, as reporters, has shown that the RpoE activity is regulated in a growth phase-dependent manner, increasing its activity as the growth rate decreases (e.g., upon entry into stationary phase). In this condition, ppGpp levels are known to increase. In vivo experiments using mutant strains that cannot produce ppGpp (*relA* and *spoT* mutants), a *rseA* mutant background, and combinations of these, have shown the requirement of ppGpp for this response, and that it is distinct from the stress signaling pathway controlled by RseA. In addition, further in vitro experiments have shown a positive regulation of some RpoE-dependent promoters by ppGpp and DksA in multi-round transcription assays from the *rpoH* P3 promoter, pointing out that this σ factor may respond to other signals apart from those indicating damage of the outer membrane or compromising the integrity of the cell envelope. Such regulation would serve to adjust RpoE levels and activity to meet cellular needs independently of RsaA [102].

As part of a recent study carried out by Gracjana Klein and collaborators [104], and which focused on peptidyl-prolyl *cis/trans* isomerases (PPIases, which catalyze *cis/trans* proline isomerization in all organisms), their enzymatic contribution and their substrates in *E. coli*, a strain lacking all six cytoplasmic PPIases (Δ*6ppi*), was constructed and analyzed. This *cis*/*trans* proline isomerization is often a rate-limiting step in protein folding, hence the importance of these folding catalysts in accelerating this reaction. A Δ*6ppi* mutant strain, among other pleiotropic phenotypes, is not viable when growing on rich medium, at a fast growth rate, and presents severe defects in protein folding. This has allowed the identification of proteins significantly affected by this catalysis, which suffered aggregation in the mutant. Pull-down experiments where four PPIases and their derivatives, either affected in their predicted active site or substrate-binding domains, were individually overexpressed in the Δ*6ppi* mutant and purified have identified RpoE and RseA as targets of the PPI FkpB, which implies another level of post-transcriptional regulation.

Interestingly, using a multicopy suppressor approach to identify factors that are limiting in the Δ*6ppi* strain led to the identification of the transcription factor DksA (among other factors). An overexpression of DksA in the Δ*6ppi* mutant background under non-permissive growth conditions is able restore growth to the strain. However, this phenotype seems to require the correct levels (wild-type levels) of GroL/S and RpoE. Intriguingly, purified DksA also has PPIase activity comparable to FkpB in vitro, which may justify its ability to restore the growth defects in the Δ*6ppi* background [105].

### 6.2. EcfG as the GSR Regulator

In Alphaproteobacteria, the Group IV σ factor EcfG, belonging to the ECF15 subgroup, plays a central role in the regulation of the so-called general stress response. This is a protective response elicited by a wide variety of unrelated stresses [106]. It may be activated by a single challenging stimulus (carbon starvation, high or low temperature, high osmolarity or oxidative stress, among others) but generates cross-protection against a whole range of them. The stationary growth phase, as a mixture of different stresses, is a representative example of a GSR-activating situation. In contrast to the specific responses, the GSR regulon is very large, affecting the expression of many different genes, which together ensures this broad range of protection. The Alphaproteobacteria represent an exception to the Proteobacteria, since they do not harbor any stationary-phase-induced RpoS homolog encoded in their genome that could control the GSR, as in the other proteobacterial groups. In turn, they use a different sigma factor and trigger a distinctive regulatory cascade based on a partner-switching mechanism to activate this response.

**Figure 6:**
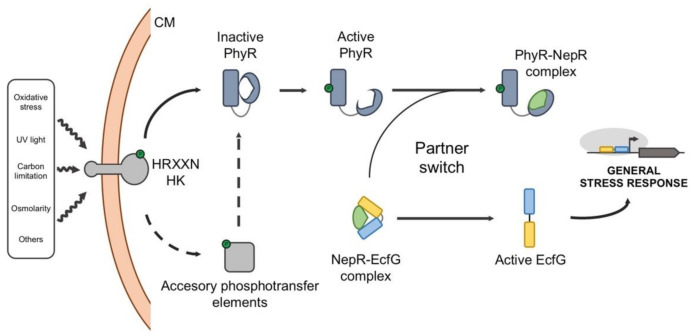
Regulatory model of the general stress response in Alphaproteobacteria. In the absence of stress, EcfG forms a complex with the anti-σ factor NepR, preventing the activation of the GSR regulon. When some kind of stress appears in the environment, it is sensed by a HRXXN histidine kinase (HK), which autophosphorylates at a His residue and transfers the phosphoryl group to an Asp residue in the response regulator PhyR. Alternatively, the HK may transfer the phosphoryl group to other accessory phosphotransfer elements, such as bifunctional kinases/phosphatases or phosphotransferases, which help to integrate signals from different origins and eventually phosphorylate PhyR in a fine-tuned manner. Upon phosphorylation, PhyR shows a σ-like domain that promotes the partner switch by sequestering NepR. This releases EcfG from inhibition, which is now available to drive the transcription of the GSR regulon.

In this pathway, three main elements are needed to transduce the signal and activate the response: the EcfG σ factor, which drives the transcription of the GSR regulon, its cognate NepR anti-σ factor, which prevents the EcfG function under non-adverse conditions, and the PhyR response regulator, which mimics EcfG structure, acting as an anti-anti-σ factor. Apart from this, stress-responsive histidine kinases and/or phosphatases regulate the activation of the GSR by modifying the phosphorylation state of PhyR [106] (Figure 6).

Although the partner-switching regulation was initially identified in the methylotrophic bacterium *Methylobacterium extorquens* [106,107,108,109], numerous genetic and biochemical studies in different species have revealed the molecular basis of the GSR regulation in the Alphaproteobacteria. In the absence of stress signals, NepR titrates EcfG, keeping it inactive, as explained in previous sections. Under stress conditions, the corresponding sensor kinases dimerize and autophosphorylate at a conserved His residue. Then, in a canonical GSR pathway, the phosphoryl group is transferred to an Asp residue in the PhyR receiver domain (PhyR^P^). This phosphotransfer triggers a conformational change in PhyR through which an N-terminal σ-like domain is exposed. This domain, mimicking the true σ factor EcfG, is able to sequester NepR in a more stable complex, thereby releasing EcfG to activate its regulon. This mechanism is possible due to the differences in the interaction kinetics between the pairs NepR-EcfG, NepR-PhyR and NepR-PhyR^P^ [22]. Once the stress is over, dephosphorylation of PhyR by different means reverses the switch, thus interrupting the GSR [110,111].

PhyR, NepR and EcfG are generally encoded at the same locus, with *nepR* and *ecfG* co-transcribed in the same operon and *phyR* transcribed divergently in another transcriptional unit. The expression of both transcriptional units is driven by GSR-dependent promoters, thus amplifying the GSR once it is activated. Additionally, a sensor or signal transducing histidine kinase (HK) coding gene (which would contain a characteristic HRXXN motif) is found in the vicinity. This type of HK specifically starts the phosphosignaling of the alphaproteobacterial GSR, and although one of them is usually encoded nearby the rest of the regulators, there might be other HRXXN HK-coding genes distributed throughout the genome [106,112,113]. Presenting a variety of sensor elements may help to specifically recognize a stimulus and modulate the activation of the GSR, resulting in an attuned output. It has been suggested that this autoregulation of all the regulators would increase the dynamic range of the response, while maintaining the stoichiometry of proteins that allows a rapid shutdown [62]. However, this classical organization may diverge in more complex scenarios, since different alphaproteobacterial species may possess a varying number of GSR regulator paralogs. Moreover, additional kinases, phosphatases and phosphotransfer elements may participate in modulating the phosphorylation state of PhyR [111,112,114], or even regulating PhyR stability [115], thus fine-tuning the level of activation of the response.

The presence of EcfG paralogs has been studied in a few organisms [74,76,116,117], and much effort has been made trying to establish possible regulatory differences among them, either by transcriptomic studies or at the level of gene expression phenotype. In spite of this, there is no general consensus about possible specific functions so far.

**Figure 7:**
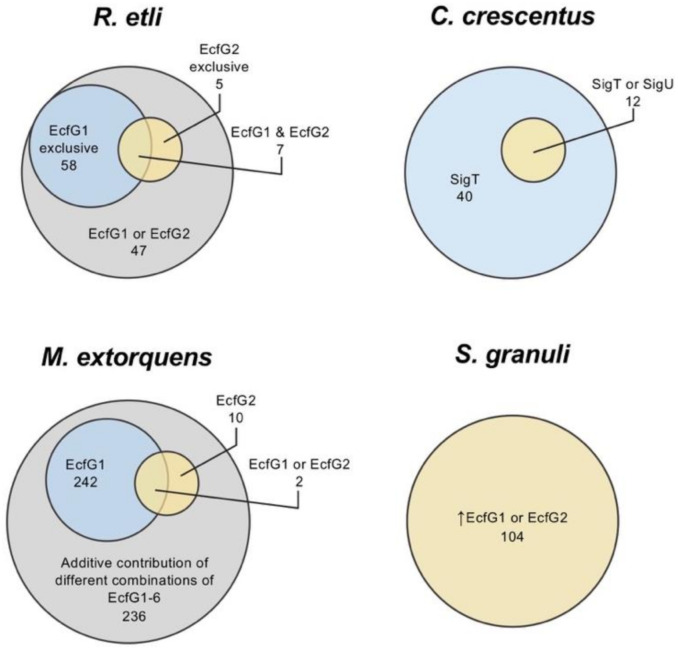
Representation of the contribution of paralogous EcfG σ factors to the activation of the GSR regulon in different Alphaproteobacteria (*R. etli*, *C. crescentus*, *M extorquens* and *S. granuli*) in which there is more than one EcfG representative. Up arrow indicates high levels of protein product. The number of regulated genes and their transcriptional dependency are based on available transcriptomic studies [74,76,116,117].

For instance, the *Rhizobium etli* genome encodes two EcfG proteins (EcfG1, encoded on the chromosome, and EcfG2, encoded on a plasmid), and although both σ factors share some target genes, they show a functional specialization controlling different sets of genes with distinct promoter sequences [76]. This originated the distinction between canonical EcfG σ factors (being autoregulated and targeting promoters with an AAC in the -35 box and a CGTT in the -10 box) and non-canonical targeting promoters with a pattern yet to be determined and without autoregulation [76]. The mutation of each paralogous *ecfG* coding gene presents different phenotypes in *R. etli*. For example, an *ecfG1* mutant shows increased heat sensitivity, while deletion of *ecfG2* accounts for reduced oxidative stress resistance [76]. In *Caulobacter crescentus*, two EcfG paralogs are present (SigT and SigU), where *sigU* is a target of the main regulator SigT and controls the expression of a small subset of genes belonging to the regulon of the SigT, indicating some kind of redundancy [118]. In *M. extorquens*, up to six of these σ factors, with unclear functional relationship, have been described, although one main regulator (EcfG1) has been identified [117]. Additionally, the EcfG2 paralog has a relevant function in activating a number of genes by itself or in concert with EcfG1. However, transcriptomic analyses suggest that the majority of the GSR regulon in *M. extorquens* (490 genes) is transcribed by an additive contribution of different combinations of the six EcfG paralogs encoded in this species (EcfG1-6). In *S. granuli* TFA, with EcfG1 and EcfG2, differential functions between the two paralogs have been detected by in vitro transcription assays, showing that although they seem to share the same target genes, EcfG2 produces higher levels of transcription than EcfG1, regardless of the promoter, and seems more important to induce GSR [74]. However, *ecfG1* transcription is strongly induced by EcfG2 under stress conditions, reaching sufficient protein levels to activate target genes by itself. The relationships between the different EcfG paralogs regarding their contribution to the activation of the GSR regulon in the aforementioned species are represented in Figure 7. More complexity is added to the core cascade when upstream regulators are also duplicated, as is the case of *S. meliloti*, which presents two PhyR (RsiB1/B2) and two NepR (RsiA1/A2) coding genes, with RsiA1 being essential [119]. In these cases, exploring the interaction kinetics among the regulatory elements is fundamental to understanding the signal transduction cascade and the role of each GSR regulator in different species.

## 7. Future Perspectives and New Insights

Among the mechanisms to respond to stress displayed by bacteria, ECFs have a significant representation, due to their diversity and abundance. Their simple, yet efficient, mode of action enables them to exert a quick and specific transcriptional regulation. Additionally, their characteristic functional regulation and target promoters seem to be conserved within each of the distinct ECF groups as is clear based on their classification, their phylogenetic relationships and genomic context conservation. Although not all ECFs respond to stress, a prominent proportion of them appear to be involved in protecting against damage coming from the inside or the outside of the cell. Some of these responses to a particular stress are narrow, involving a limited number of operons, but other responses are very broad (e.g., the GSR), implying the induction of many genes presumably involved in coping with different stresses that may appear together in natural conditions. A well-characterized example of these multi-stress conditions is the stationary phase of growth, which is known to activate the GSR [106]. Especially in the case of broad stress responses, such as the aforementioned stationary phase, a cross-protection for different stresses than the one that elicited the response in the first place can be generated [75,107,108,119,120,121]. On the other hand, there are examples of different stresses causing similar effects, thus converging in one sole response against them. This is the case for RpoE that responds to cell envelope integrity alterations produced by heat shock or issues with the cell envelop biosynthesis or the assembly of OMPs and LPS [95,122,123,124,125,126].

The extensive use of transcriptomics and bioinformatics have allowed the definition of many ECF regulons related to stress responses. Among their target genes, many well-known elements that protect against specific stresses are found. A plethora of examples can be found in the literature, such as catalases and peroxiredoxins to protect against oxidative stress [3,4], chaperones and proteases to mitigate the effects of temperature shifts and organic solvents [1], outer membrane proteins to maintain the cell envelop integrity [2] or DNA repair systems to fix lesions in the genomic DNA [74,127,128]. However, and most intriguingly, a remarkable proportion of target genes retrieved from transcriptomic analyses still have an unknown function (for instance, in the direct GSR regulon of the alphaproteobacterium *S. granuli*, only 37% of the genes have an annotated function [74]). These unknown genes may represent redundant mechanism to protect against the already known conditions that are hostile for bacterial growth or may be necessary to respond to untested forms of stress. Additionally, these genes might be involved in responses to biotic stresses in nature [129], such as competition with other organisms, which have no relevance in axenic laboratory cultures and remain understudied with respect to specific gene functions. Further characterization of target gene functions is crucial to fully understand what bacteria sense as stress and the gene repertoire they have to successfully cope with either abiotic or biotic stress conditions.

The wide variability of the ECFs as a protein family is reflected in the recent expansion of their classification. On the base of updated protein and genome databases, the reclassification built by Casas-Pastor et al. accurately shows the variety of potential modes of regulation, target promoters and phylogenetic relationships of this group of proteins [56]. Furthermore, this classification could expand even further, which is anticipated by the number of unclassified ECFs that resulted from this analysis and the continuous update of the bacterial genome databases. However, part of the diversity of the ECF group, mainly that on the edge of the ECF definition, is missing in the classification due to the limitations of the methodology employed for its construction. For example, ECFs encoded in viral genomes and ECFs whose sequence diverges significantly compared to the bona fide ECF sequences could not be included in the analysis, which makes the classification still amenable to further development.

Regarding their biological roles in bacterial gene regulation, putting the focus on the function of their target genes, we have attempted to ascribe a broad function to each of the ECF groups, based on the knowledge about experimentally studied representatives. Basically, we have differentiated between those ECFs involved in protecting the cell against internal or external threats and maintaining its integrity (45 groups) and those implicated in nutritional, metabolic and biosynthetic processes (five groups), with some groups comprising representatives that could fit in both of these classes (five groups). The classification has revealed not only that the way of regulation and the target operons within each group seems to be homogeneous, but also the importance of ECFs in the protection of the cell, since most of the documented ECF systems belong to the first class. Moreover, and most intriguing, the majority of the ECF groups defined in the latest classification do not have any experimentally studied representative (102 groups), and thus, their putative biological role is still unaddressed. This gives an idea of the vast universe of potential information (regulatory mechanisms, target promoters, biological responses, gene functions, etc.) that is yet to be explored, and that will challenge molecular microbiologists in the future, even beyond the stress responses.

Conventional transcriptional regulators are, in general terms, DNA-binding proteins that control (positively or negatively) transcription after providing a certain conformation to the promoter region and/or facilitating or impeding the anchoring of the RNAP and the transcriptional initiation [130]. Unlike them, ECFs are essential for the transcription of their target genes, in a sense that, unless there were some functional redundancy, the absence of an ECF would completely abolish the transcription of its target genes. This allows a tight and rapid gene regulation, but also requires a tight control of the ECF activity. There are different mechanisms to exert this control, although the most widespread is the negative regulation by an anti-σ factor. This results in highly specific σ-anti-σ interactions, which is reflected in the orthogonality of these systems [131] and, for example, in the hypothesized co-evolution of the interacting surfaces of ASDIs and their cognate ECFs. This co-conservation has been proposed to apply for other types of σ-anti-σ pairs due to the similarity of the binding modes in spite of the poor sequence conservation [61]. However, this orthogonality may be limited, and non-cognate σ-anti-σ cross-talks might have a biological significance in vivo [132]. Nonetheless, there is still a lot to explore about other ECF regulatory mechanisms. STPK-regulated ECFs and representatives with C-terminal extensions have just started to be characterized and, apart from predictive in silico data, no mechanistic information has been obtained yet about the regulation by N-terminal extensions. Furthermore, ECF representatives with no apparent anti-σ factor (or other regulators) encoded in the vicinity still raise questions: are all ECFs encoded nearby the genes encoding their regulators? Are there other regulatory mechanisms for ECFs that remain unknown? Are there ECFs controlling their activity without the participation of any other regulator or regulatory extension? There is already the example of the ECF SigK, from *M. tuberculosis*, whose activity is controlled by two Cys residues that form a disulphide bond depending on the presence of oxidants [133]. Nevertheless, the activity of SigK is also controlled by the anti-σ factor RskA. This is not the only example of an ECF controlled by more than one mechanism [134], and based on sequence and synteny analyses, the multi-level control of the ECF activity might be more common than expected, giving an idea of the signal integration potential of ECFs [56].

Due to the aforementioned properties that distinguish them from conventional transcriptional regulators, σ factors have received increasing interest to be used in engineered heterologous expression systems, using different approaches for their optimization [135,136]. ECFs in particular have the advantage that their transcription initiation mechanism is based on the minimal number of elements that a σ factor needs (i.e., the σ_2_ and the σ_4_ domains), which makes them, together with their target promoters, amenable to be more easily engineered. That race has already started and, for instance, different ECF-promoter partners from different bacterial species and belonging to distinct ECF groups within the classification have been optimized, even forming regulatory cascades, in heterologous systems for *E. coli* and *B. subtilis* [137]. Using a different approach, a rational screening was performed previously to find ECF-promoter and ECF-anti-σ pairs showing the highest orthogonality in *E. coli* [131]. In it, two representatives of each ECF “major” group (86 in total) of the original classification (the only available at that time) were paired with a library of more than 700 promoters to evaluate their specificity, resulting in 20 ECF-promoter pairs exhibiting high orthogonality. That set of pairs could even be expanded by shuffling their σ_2_ and σ_4_ domains and their cognate -10 and -35 boxes. A similar rationale was followed to find highly orthogonal ECF-anti-σ pairs, and although specific pairs could be found, the cross-reactivity levels appeared higher than in the ECF-promoter pairs (which is coherent with posterior ECF-anti-σ orthogonality and its limits [132]).

The current methods for a priori target promoter determination of ECFs is limited, since it is based on previously known ECF-promoter pairs and predictions on autoregulated ECFs, comprising only a part of the ECF protein group. Due to this, experimentally studying the ECF-promoter specificity requires high-throughput approaches such as that followed by Rhodius et al. [131]. However, a recent study has shed light on the code behind the ECF-promoter interaction [50]. Using a set of autoregulated ECFs, whose target promoters are known, they have established the position-specific relationship between the nucleotides in the -10 and -35 boxes and the residues within the respective ECF that interact with them. This effort for matching specific residues in the σ_2_ and σ_4_ domains of ECFs to their most likely cognate nucleotides in the corresponding -10 and -35 boxes has allowed the de novo prediction of the target promoter for nearly 70% of ECFs. Promisingly, this opens the door to design and engineer ECF-promoter systems à la carte in the future.

## Supplementary Materials

The following are available online at https://www.mdpi.com/1422-0067/22/8/3900/s1.
